# A Case of Strangulated Inguinal Hernia Mistaken for Bilious Colic by a Thompsonian—Consequent Death

**Published:** 1849-01

**Authors:** R. Horsely

**Affiliations:** Medical Student


### ARTICLE VI.

*A Case of Strangulated Inguinal Hernia mistaken for Bilious Colic by a Thompsonian—Consequent Death.* By R. HORSELY, medical student.

Mr. H——, a young man æt. 18, a day laborer, of robust health and strong constitution, has had for several years a reducible inguinal hernia. On Friday, Oct. 6, while carrying a weight upon his shoulder, the protruding intestine came down into the scrotum, and swelled to an enormous size. This was attended with violent pain, not only in the part, but all over the abdomen; sickness, vomiting of fœcal matter, suppression of stools, fever, &c. His symptoms gradually grew worse, and on the following day a *Thompsonian* was called in. He assured the patient that "he could help him without any difficulty, and that his disease was the bilious colic."

The quack proceeded to apply *Cataplasms* and other fomentations to the abdomen, without discovering the hernia. He gave, internally, large quantities of cayanne, both in powder and tincture, soot tea, and, according to his own story, seven portions of physic, besides other mixtures. Finding his efforts unavailing, and still desirous of procuring an evacuation from the bowels, he left the patient to procure a bottle of Croton oil, with a promise to call again and complete his design. In the mean time, the strangulated intestine mortified, and the patient died, thirty-six hours after the accident. The young man's disease was discovered after death by an attendant who wrapped him in his winding-sheet and helped to perform the last rites to this unfortunate victim of quackery. In a day or two after, the attendant met with the quack and asked him if he knew what was the matter with the young man. He replied that "his disease was the bilious colic." The attendant assured him that it was entirely a different thing—that "the young man had a burst, and his bag was swelled larger than his fists, that the intestines were mortified," &c. The quack appeared to be confounded, and said that "he was very sorry, thet he did not know that that was the case, for he called himself first-rate in such diseases as rupture," &c. He boasted of what he had done, and what he could do: a general and sure mark of an empiric. This is one among the large number of lives that are being daily sacrificed to the ignorant and vile absurdities of quacks.

